# Regulation of Syntaxin3B-Mediated Membrane Fusion by T14, Munc18, and Complexin

**DOI:** 10.3390/biom13101463

**Published:** 2023-09-28

**Authors:** Rajkishor Nishad, Miguel Betancourt-Solis, Himani Dey, Ruth Heidelberger, James A. McNew

**Affiliations:** 1 Department of BioSciences, Rice University, 6500 Main Street, MS 601, Houston, TX 77005, USA; ma.betancourtsolis@gmail.com; 2 Lonza Biologics, 14905 Kirby Dr, Houston, TX 77047, USA; 3 Department of Neurobiology and Anatomy, McGovern Medical School, The University of Texas Health Science Center, Houston (UTHealth Houston), 6431 Fannin Street, Houston, TX 77030, USA; himani.dey@uth.tmc.edu

**Keywords:** STX3B, syntaxin, ribbon synapse, Munc18, complexin

## Abstract

Retinal neurons that form ribbon-style synapses operate over a wide dynamic range, continuously relaying visual information to their downstream targets. The remarkable signaling abilities of these neurons are supported by specialized presynaptic machinery, one component of which is syntaxin3B. Syntaxin3B is an essential t-SNARE protein of photoreceptors and bipolar cells that is required for neurotransmitter release. It has a light-regulated phosphorylation site in its N-terminal domain at T14 that has been proposed to modulate membrane fusion. However, a direct test of the latter has been lacking. Using a well-controlled in vitro fusion assay, we found that a phosphomimetic T14 syntaxin3B mutation leads to a small but significant enhancement of SNARE-mediated membrane fusion following the formation of the t-SNARE complex. While the addition of Munc18a had only a minimal effect on membrane fusion mediated by SNARE complexes containing wild-type syntaxin3B, a more significant enhancement was observed in the presence of Munc18a when the SNARE complexes contained a syntaxin3B T14 phosphomimetic mutant. Finally, we showed that the retinal-specific complexins (Cpx III and Cpx IV) inhibited membrane fusion mediated by syntaxin3B-containing SNARE complexes in a dose-dependent manner. Collectively, our results establish that membrane fusion mediated by syntaxin3B-containing SNARE complexes is regulated by the T14 residue of syntaxin3B, Munc18a, and Cpxs III and IV.

## 1. Introduction

Retinal neuron photoreceptors and bipolar cells continuously transmit visual information over many log units of dynamic range at specialized active zones called ribbon-style active zones. The remarkable signaling abilities of these neurons are supported by unique presynaptic machinery, one component of which is syntaxin3B [1,2,3,4,5]. Syntaxin3B (STX3B) is a retinal-specific t-SNARE (soluble *N*-ethylmaleimide-sensitive factor attachment protein receptor) protein [3,4,6], and like the canonical syntaxin1 of conventional synapses, it forms a tripartite SNARE complex with SNAP25 (Synaptosome Associated Protein 25) and VAMP2/synaptobrevin2 (Vesicle-associated membrane protein 2) to comprise the core machinery required for membrane fusion [1,3,4]. Consistent with its role as a synaptic SNARE protein, exocytosis from photoreceptor and bipolar cell synaptic boutons is inhibited when syntaxin3B is prevented from interacting with its cognate SNARE binding partners [4,5]. Furthermore, in mouse models and human subjects, loss of syntaxin3B functionality results in retinal degeneration and vision loss [6,7].

In addition to its essential role in neurotransmitter release at retinal ribbon-style synapses, syntaxin3B has also been suggested to have a modulatory role in exocytosis [8,9]. At conventional synapses, Munc13 is required for the transition of syntaxin1A from a closed conformation that is unable to participate in SNARE complex formation to an open conformation that is permissive for SNARE assembly [10,11,12]. While syntaxin3B also has closed and open conformations [9,13], neurotransmitter release from photoreceptors does not appear to require Munc13 [14]. Rather, syntaxin3B has a unique CaMKII-dependent phosphorylation site in its N-terminus at T14 [9,15] that is regulated by natural stimuli in vivo [8,9]. The T14E syntaxin3B phosphomimetic mutant (syntaxin3B(T14E) binds SNAP25 to form a t-SNARE complex more readily than wild-type syntaxin3B [9], suggesting that phosphorylation of syntaxin 3B at T14 favors the syntaxin3B open conformation [8,9]. This hypothesis has been confirmed by a recent FRET-based study in which syntaxin3B(T14E) was found to sample the syntaxin3B open state, whereas wild-type syntaxin3B was primarily in the closed conformation [13].

Subtle changes in the N-terminal domain of syntaxin, such as by phosphorylation, are predicted to alter a critical interaction between syntaxin1 and Munc18, a molecular chaperone of SNARE complex formation [16,17,18,19,20]. Munc18 binds to the N-terminal domain of free syntaxin3B, where it stabilizes the syntaxin closed configuration and, upon activation of syntaxin, facilitates the assembly of SNARE complexes [13,21,22,23,24,25]. Retinal ribbon-style synapses utilize Munc18-1/Munc18a [26,27,28], and Munc18a has been shown to bind free syntaxin3B and to syntaxin3B-containing complexes [9,29,30]. At conventional synapses, loss of Munc18a function results in severe impairment [31,32,33], while a Munc18-1 mutation with altered syntaxin3B binding has been linked to a vision disorder in human subjects [29].

Complexins are small protein molecules (14–20 kDa) that interact with the zippering(ed) SNARE complex and aid in the control of neurotransmitter release [34,35]. At conventional synapses, Cpx I and Cpx II govern a late step in the membrane hemifusion, most likely by stabilizing SNARE complexes and maintaining synaptic vesicles in a highly release competent state [34,35,36,37]. By contrast, ribbon-style synapses of retinal photoreceptors and bipolar cells express two different members of the Cpx superfamily, Cpx III and Cpx IV. These complexins exhibit unusual structural and functional characteristics [38]. While the precise mechanism in which they operate at retinal ribbon-style synapses has not yet been confirmed, interference with Cpx III and IV function has been suggested to play roles in the adaptation and fidelity of synaptic signaling [39,40,41,42].

In this study, we examined syntaxin3B-mediated membrane fusion using a well-characterized lipid mixing assay [25,43,44,45] and probed the potential regulation by T14, Munc18a, and Cpxs III and IV. We found that the phosphomimetic T14E mutation of syntaxin3B modestly augmented fusion, suggestive of a regulatory role for T14 of syntaxin3B on SNARE-mediated membrane fusion following preassembly of the t-SNARE complex between syntaxin3B and SNAP25. Munc18 bound to SNARE complexes containing syntaxin3B, and this was not altered by a phosphomimetic T14 syntaxin3B mutation. However, Munc18 enhanced membrane fusion mediated by SNARE complexes containing the T14E syntaxin3B phosphomimetic mutation to a greater extent than those containing wild-type syntaxin3B, suggesting an increase in functionality in the presence of the T14E syntaxin3B mutation. These results raise the possibility of a modulatory role for T14 downstream of SNARE complex formation in addition to its presumptive role in facilitating syntaxin3B activation. Finally, our results demonstrate an inhibitory role of Cpxs III and IV on membrane fusion mediated by syntaxin3B-containing SNARE complexes, consistent with a clamping function similar to that suggested for other family Cpx members at conventional synapses [46,47,48]. Collectively, our results establish the importance of the T14 residue of syntaxin3B, Munc18a, and Cpxs III and IV in the regulation of membrane fusion at retinal ribbon-style synapses. Furthermore, given that the N-terminal domain, including T14, is conserved between syntaxin3B and the more ubiquitously expressed syntaxin3A [3,6,9], our results have the potential to enhance our understanding of membrane fusion and its regulation in cell types beyond specialized retinal neurons.

## 2. Materials and Methods

### 2.1. Molecular Biology

The Syntaxin 3B full-length expressing construct (pJM485) previously described [3] was used for making recombinant Syntaxin3B. The pFP247 clone, which encodes for SNAP25b, and the pTW38 clone, which encodes VAMP2, were used as previously described [49].

### 2.2. Site-Directed Mutagenesis

The Syntaxin3B mutant (T14E) was made using the primer (GCTGAAGGCCAAGCAGCTGGAACAGGATGATGACACGGACG) and its complementary primer. Similarly, Syntaxin3B mutant (T14A) was made with (CTGAAGGCCAAGCAGCTGGCACAGGATGATGACACGGAC) and its corresponding complementary primer. All point mutants of Syntaxin3B were made by PCR using a QuikChange II site-directed mutagenesis kit (Agilent, Santa Clara, CA, USA).

### 2.3. Protein Expression and Purification

#### 2.3.1. Syntaxin 3B and His_6_SNAP-25

Syntaxin 3B and His_6_SNAP-25, vectors pJM485 and pFP247, were cotransformed into *E. coli* strain BL21(DE3) and coexpressed. Four liters of *E. coli* [BL21(DE3)] were grown at 37 °C in SuperBroth (TekNova) to an OD600 between 0.6 and 0.8. Protein expression was induced with 0.25 mM IPTG while shaking for 4 h at 37 °C at 200 rpm. Cells were harvested by pelleting for 15 min at 7500 rcf at 4 °C on an Avanti JHC centrifuge with a JS-5.0 rotor. The cell pellet was resuspended in A200 (25 mM HEPES (pH 7.4) and 200 mM KCl). Cells were then centrifuged for 5 min at 4 °C at 10,950 rcf in a JA-10 rotor. Cells were then resuspended in 40 mL of breaking buffer (A200 plus 10% glycerol, 2 mM 2-mercaptoethanol, 4% Triton X-100, 40 mM imidazole, and one Complete protease inhibitor mixture tablet (Roche Applied Science). Cells were then passed three times through an EmulsiFlex C3 high-pressure homogenizer (Avestin, Ottawa, ON, Canada)) at 15,000–20,000 p.s.i. Cell debris was then removed by centrifugation in a JA20 rotor for 30 min at 8000 rpm. Cell extracts were then cleared by centrifugation at 125,000 rcf at 4 °C using a Type 45-Ti rotor in an Optima LE-80K ultracentrifuge. Cleared lysate was filtered through a 0.45-m pore cellulose nitrate sterile membrane filter (Whatman). The extract was passed through an equilibrated (A100 (25 mM HEPES (pH 7.4) and 100 mM KCl) + 10% glycerol, 2 mM 2-mercaptoethanol, 1% Triton X-100, 40 mM imidazole) HiTrap Chelating HP column (GE Healthcare) in the ÄKTA prime chromatography system (Amersham Biosciences, Piscataway, NJ, USA) to bind SNAP25B and co-bound Syntaxin3B.

The column was then washed with 25 mL of A100 buffer + 10% glycerol, 2 mM 2-mercaptoethanol, 0.1% Anapoe X-100, and 40 mM imidazole. Protein was then eluted in a 30-mL linear gradient of imidazole from 40 to 500 mM and a final 5 mL wash at 500 mM. Peak fractions were pooled, aliquoted, and flash-frozen in liquid nitrogen and stored at −80 °C. Protein was quantified by Amido Black assays [50], and yields ranged from ∼2 to 4 mg/mL. Also, a 5 μL protein sample was run on 12% SDS-PAGE and Coomassie-stained to confirm the protein purity.

#### 2.3.2. VAMP2

VAMP2-His_6_ (M. musculus vector pTW38) and VAMP2ΔTMD (pJM513) were transformed and expressed into *E. coli* strain BL21(DE3) and protein purification done as described previously [51]. The protein yields were ∼1 to 2.5 mg/mL as determined by Amido Black assays.

#### 2.3.3. Munc18a

Munc18a-His_6_ (*R. norvegicus*) was produced by expressing pJM546 (plasmid received from Dr. Jingshi Shen, University of Colorado at Boulder) in BL21 (DE3) *Escherichia coli* (Stratagene). Munc18a-His_6_ purification was performed as described previously [23]. Briefly, cells were grown at 37 °C in 4 L of SuperBroth (Teknova, Half Moon Bay, CA, USA) to an OD600 of 0.6 to 0.8 while shaking at 200 rpm. Protein expression was induced with 1 mM IPTG while shaking 4 h at 37 °C at 200 rpm. The cell pellet was resuspended in lysis buffer and passed through an Emulsiflex-C5 High-Pressure Homogenizer (Avestin, Ottawa, ON, Canada) and lysed by pressure. Cell debris was then removed by centrifugation in a JA20 rotor for 30 min at 8000 rpm. Cell extracts were then cleared by centrifugation at 125,000 rcf at 4 °C using a Type 45-Ti rotor in an Optima LE-80K ultracentrifuge. The extract was then passed over a HiTrap HP Ni2^+^-chelating column (GE Healthcare, Waukesha, WI, USA) in the ÄKTA Prime chromatography system (Amersham Biosciences, Piscataway, NJ, USA) to bind Munc18a-H_6_. The column was washed with 10 column volumes of buffer A150 (25 mM HEPES, pH 7.4, 150 mM KCl, 10% glycerol, and 2 mM β-mercaptoethanol [BME]). Protein was eluted in 20 column volumes with a linear gradient of 20 to 500 mM imidazole in buffer A150. Peak fractions were pooled, aliquoted, and flash-frozen in liquid nitrogen and stored at −80 °C. Protein was quantified by Amido Black assays [50], and yields ranged from ∼7 to 9 mg/mL. To confirm the protein purity, a 5 μL protein sample was run on 10% SDS-PAGE and Coomassie-stained.

#### 2.3.4. Complexin III and IV

We produced His6-Mm_Cpx III (clone pJM 480) and His6-Mm_Cpx IV (clone pJM 393) by expression of in Bl21(DE3) *Escherichia coli* strain (Novagen). Cells were grown to OD600 0.6–0.8 in 4 L SuperBroth (Teknova, Half Moon Bay, CA, USA). Expression was induced with 0.25 mM IPTG for 4 hrs at 200 rpm shaking incubator at 37 °C. The cell pellet was resuspended in lysis buffer and passed through an Emulsiflex-C5 High-Pressure Homogenizer (Avestin, Ottawa, ON, Canada) and lysed by pressure. Cell debris was then removed by centrifugation in a JA20 rotor for 30 min at 8000 rpm. Cell extracts were then cleared by centrifugation at 125,000 rcf at 4 °C using a Type 45-Ti rotor in an Optima LE-80 K ultracentrifuge. The extract was then passed over a HiTrap HP Ni2^+^-chelating column (GE Healthcare, Waukesha, WI, USA) in the ÄKTA Prime chromatography system (Amersham Biosciences, Piscataway, NJ, USA) to bind His6-Mm_Cpx III or His6-Mm_Cpx IV. The column was washed with 10 column volumes of buffer A150 (25 mM HEPES, pH 7.4, 150 mM KCl, 10% glycerol, and 2 mM β-mercaptoethanol [BME]). Protein was eluted in 20 column volumes with a linear gradient of 20 to 500 mM imidazole in buffer A150. Peak fractions were pooled, aliquoted, and flash-frozen in liquid nitrogen and stored at −80 °C. Protein was quantified by Amido Black assays [50], and yields ranged from ∼1 to 3 mg/mL. To confirm the protein purity, a 5 μL protein sample was run on 12% SDS-PAGE and Coomassie-stained.

### 2.4. Liposome Production and Reconstitution

Liposome production and reconstitution were done as reported previously [25,44] with the following modifications. Unlabeled liposomes constituted 1-palmitoyl-2-oleoyl-glycero-3-phosphocholine (POPC) and 1,2-dioleoyl-sn-glycero-3-phospho-L-serine (DOPS) in an 85:15 molar ratio, and labeled liposomes were constituted with POPC/DOPS/Rh-DPPE/NBD-DPPE (82:15:1.5:1.5 molar ratio). A trace amount of [3H]1-palmitoyl2-palmitoylphosphatidylethanolamine (DPPE) (American Radiolabeled Chemicals) was mixed with unlabeled and labeled liposomes to determine their concentration by scintillation counting. This choice of lipids yields very stable liposomes that are chemically inert under the conditions of this study. Lipid mixes prepared in chloroform were dried up by a nitrogen stream for 10 min and an extra 30 min in a desiccator by applying a vacuum. Next, the dried lipid film was resuspended to 10 mM in A100 buffer with 10% glycerol, 2 mM 2-mercaptoethanol, and 1 mM EDTA by gentle vortexing for 15 min at room temperature. Next, the lipid mix was freeze-thawed for 10 cycles in liquid nitrogen and then passed 19 times through a 100-nm pore polycarbonate membrane (Avanti). Syntaxin3B and VAMP2 were reconstituted by the detergent-assisted insertion method reported previously [52]. Protein, Triton X-100, A100 buffer, and lipid were incubated for 1 h at 4 °C on a nutator at a 1:100 protein/lipid ratio unless otherwise stated, and an effective detergent/lipid ratio of ~0.7, determined by *R*_eff_ = *D*_total_ − *D*_water_/[lipid], where *D*_total_ is the total detergent concentration and *D*_water_ is the monomeric detergent concentration (0.18 mM) with lipids. The solution was then added to swollen Bio-Beads SM-2 (#1523920) to remove the Triton X-100 (1 g per 70 mg of Triton X-100) and incubated overnight at 4 °C. Unincorporated protein was pelleted by centrifugation at 16,000 rcf for 10 min. Lipid concentration was then measured by scintillation counting.

### 2.5. Gradient Preparation and Flotation

Proteoliposomes were finally separated from unincorporated lipids and proteins by a three-layer discontinuous gradient as described previously [25] with the following modifications. A stock solution of 80% Nycodenz in A100 was diluted with the proteoliposomes in A100 with 10% glycerol, 2 mM 2-mercaptoethanol, and 1 mM EDTA (reconstitution buffer), to 40% (*w*/*v*) Nycodenz, to a final volume of 300 µL. This was added to a 5 × 41-mm Ultra-Clear tube (Beckman, Brea, CA, USA). The next layer was a 250 μL of 30% (*w*/*v*) Nycodenz on top of the 40% Nycodenz and proteoliposome mixture. The final layer was made with 50 μL of reconstitution buffer without glycerol. The gradient was then centrifuged in a SW55-Ti rotor for 4 h at 48,000 rpm (218,500× *g*) at 4 °C. Proteoliposomes were harvested (60 µL t-SNARE and 60 µL v-SNARE) at the 0%/30% gradient interface. Amido Black assay was performed to determine the amount of protein incorporated into the liposome.

### 2.6. In Vitro Lipid Mixing Assays

Fusion was measured by lipid-mixing assays based on the method described previously [25]. Briefly, labeled (donor) and unlabeled (acceptor) proteoliposomes were brought to a final concentration of 1.5μM in reconstitution buffer for a 60 μL reaction setup. This reaction setup was performed in a 96-well Fluoronunc Polysorp plate (Nunc, Naperville, IL, USA) and incubated overnight on nutating at 4 °C. Next, a ninety-six well plate with the reaction setup was placed in a preheated at 37 °C fluorescent plate reader (Tecan Infinite M200). Acceptor (Syntaxin3B) and donor (v-SNARE) proteoliposomes were fused in a 1:1 ratio for 2 h at 37 °C, and readings were measured every 2 min. Ten microliters of 2.5% (wt/vol) *n*-dodecylmaltoside detergent was added at 2 h to produce maximum NBD fluorescence. NBD fluorescence was normalized to the percent of maximum fluorescence.

### 2.7. Statistical Analyses

Data analyses were performed in Excel (Microsoft, Redmond, WA, USA) and in Prism 8 (GraphPad Software, Inc., San Diego, CA, USA). Pair-wise comparisons between two conditions were performed using a two-tailed student’s *t*-test. Comparison across experiments were performed using a one-way ANOVA followed by Tukey’s multiple comparisons.

## 3. Results

### 3.1. Syntaxin3B with a Phosphomimetic Mutation at T14 Enhances Membrane Fusion

In the synaptic boutons of mammalian retinal photoreceptors and bipolar cells, phosphorylation of T14 of syntaxin 3B occurs under physiological conditions in a light-regulated, Ca^2+^-dependent manner [8,9]. There is also evidence to suggest that phosphorylation of syntaxin 3B at T14 favors the transition of syntaxin 3B from a closed conformation to an open conformation, facilitating its interactions with SNAP25 [8,9,13]. However, the role of the T14 site in the regulation of membrane fusion mediated by syntaxin3B-containing SNARE complexes has yet to be demonstrated.

**Figure 1:**
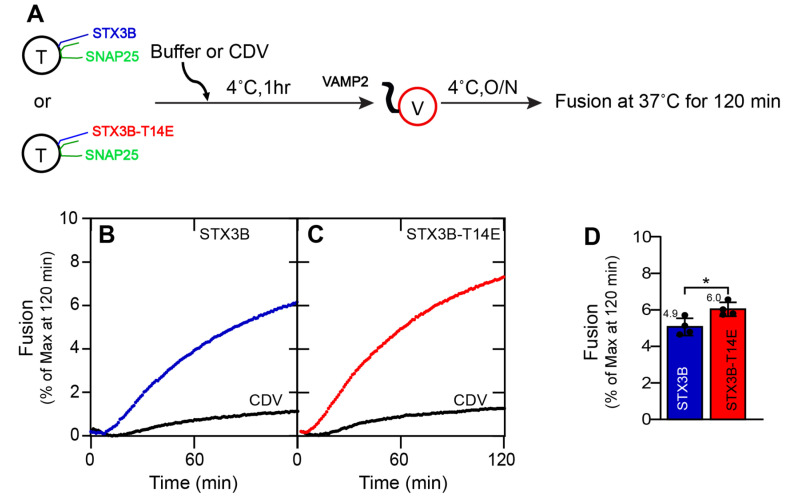
Soluble Munc18a shows a modest stimulatory role in STX3B-mediated membrane fusion. (**A**) A schematic representation of SNARE liposome fusion assays. T = t-SNARE (Syntaxin3B/SNAP25b) proteoliposome. V = v-SNARE (VAMP2) proteoliposome. CDV = Cytoplasmic domain of VAMP2. (**B**,**C**) Kinetic fusion graph of unlabeled acceptor t-SNARE proteoliposome (1.5 µM) (STX3B (**B**) or STX3B-T14E (**C**)) mixed with fluorescently labeled donor v-SNARE proteoliposome (1.5 µM) in the presence of 20 µM CDV or an equal volume of buffer (A100). The fusion assay was performed at 37 °C for 2 h after incubating overnight at 4 °C. Detergent was added at 120 min, and the relative NBD fluorescence data are represented as percent (%) of maximum fluorescence versus time. (**D**) Average fusion reaction rates at 120 min are presented as a bar graph of the reconstituted fusion assay shown in (**B**,**C**). Error bars indicate standard error mean. Data presented as mean ± SEM (*n* = 4 independent replicates). *p* values were calculated using a Student *t*-test. * *p* < 0.05.

We employed a reconstituted in vitro fusion system to probe the effects of syntaxin 3B with and without a T14E phosphomimetic mutation on membrane fusion. To this end, recombinant t-SNARE proteins were produced by co-expressing full-length syntaxin3B (or T14E) and His tagged-SNAP25b [3]. Full-length VAMP2-His_6_ was produced to perform the role of the v-SNARE. Retinal t-SNAREs, syntaxin3B/SNAP25b or syntaxin3B-T14E/SNAP25b (acceptor), and VAMP2 (donor) were reconstituted into liposomes at 1:100 surface densities [44,52]. Syntaxin3B/SNAP25b-containing proteoliposomes were mixed with VAMP2-containing proteoliposomes in a 1:1 ratio at the concentration of 1.5 µM for each SNARE. Fusion was measured using a well-characterized fluorescence resonance energy transfer (FRET) lipid mixing assay between t-SNARE and v-SNARE-containing liposomes (Figure 1A) [25]. A soluble version of VAMP2 (CDV) served as a negative control (Figure 1B,C) [43].

Results revealed a small but statistically significant improvement in membrane fusion driven by the SNARE complexes containing the syntaxin3B phosphomimetic T14E mutation when compared to SNARE complexes containing wildtype syntaxin3B (Figure 1C,D; STX3B: 4.91 ± 0.15; STX3B(T14E): 6.04 ± 0.2; *n* = 4; *p* = 0.005). Given that the t-SNARE complex between syntaxin3B and SNAP25 is preassembled prior to reconstitution [24], this result suggests that T14, located in the N-terminus of syntaxin3B, has a small modulatory role in membrane fusion after t-SNARE preassembly.

### 3.2. Munc18a Interacts with Membrane-Bound t-SNARE Complexes Containing Syntaxin3B

**Figure 2:**
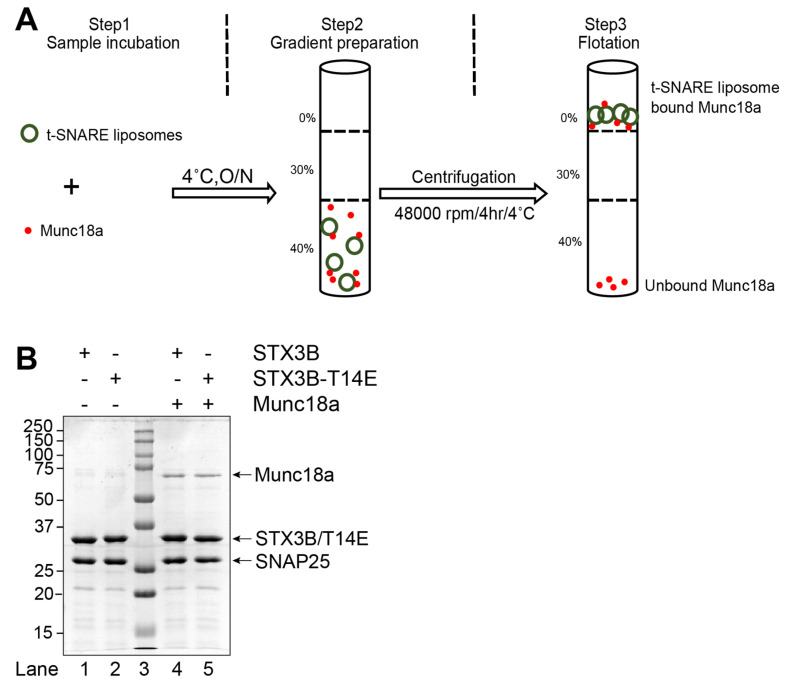
Munc18a functionally interacts with the assembled wild-type (Syntaxin3B/SNAP25b) and mutant (Syntaxin3B-T14E/SNAP25b) SNARE complex. (**A**) A schematic representation of Munc18a binding to assembled t-SNARE complex (Syntaxin3B/SNAP25b). t-SNARE liposome (5 µM) and soluble Munc18a (15 µM) were incubated for overnight at 4 °C. Accudenz gradient preparation and centrifugation were performed to obtain Munc18a bound SNARE complex. (**B**) Coomassie blue-stained SDS-PAGE gel showing the binding of Munc18a to SNARE liposome. Lanes 1 and 2 indicate input materials for Syntaxin3B/SNAP25b and Syntaxin3B-T14E/SNAP25b; Lane 3 indicates Mr = Marker, Lanes 4 and 5 indicate float-up result showing the binding of Munc18a to indicated SNAREs liposome. Ten microliters of sample were loaded per lane on 10% SDS-PAGE. The arrow indicates the respective protein band molecular weight.

We next examined the interaction of Munc18a with SNARE complexes with and without the syntaxin3B T14E mutation. We produced recombinant Munc18a in *E. coli* and determined its ability to bind to reconstituted, full-length syntaxin3B in the context of a liposome-embedded assembled t-SNARE complex. We found that Munc18a efficiently bound to t-SNARE-containing liposomes (Figure 2B, lane 4), confirming that Munc18a interacts with syntaxin3B-containing t-SNARE complexes, much like we have previously shown with syntaxin1A [24]. Additionally, we found that the syntaxin3B(T14E) mutant SNARE bound Munc18a equally well when present in a t-SNARE complex (Figure 2B, lane5), in contrast to the reduced interaction reported with free syntaxin3B [13].

### 3.3. Syntaxin3B, with a Phosphomimetic Mutation at T14, Exhibits Enhanced Membrane Fusion in the Presence of Munc18a

We next sought to determine whether the binding of Munc18a to wild-type or the phosphomimetic mutant syntaxin3B had functional consequences for in vitro fusion. Recombinant Munc18a-H6 protein was expressed in *E. coli* and directly added to t-SNARE liposomes containing wild-type or mutant syntaxin3B and SNAP25 for 1 h. Then, v-SNARE (VAMP2)-containing liposomes were added to the mix and incubated overnight.

**Figure 3:**
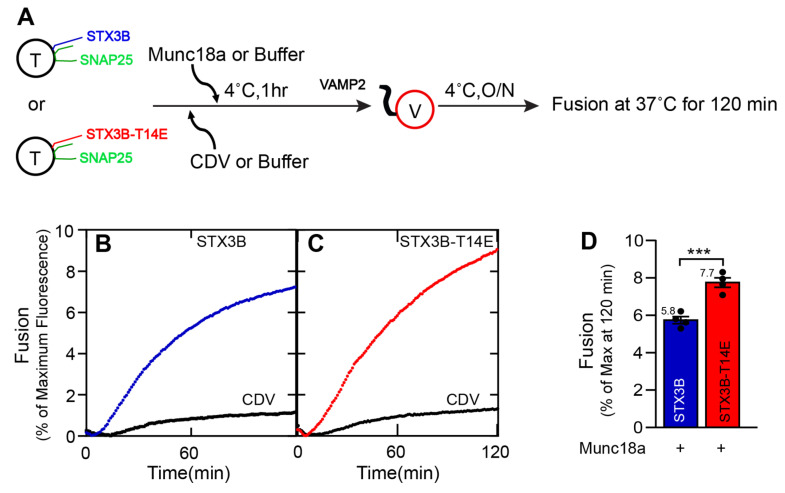
Phosphomimetic mutation of Syntaxin 3B-T14 enhances Munc18a-mediated stimulation of membrane fusion. (**A**) A schematic representation of the incubation method for mutant SNARE liposome fusion assays. T = t-SNARE (Syntaxin3B-T14E/SNAP25b) proteoliposome. V = v-SNARE (VAMP2) proteoliposome. CDV = Cytoplasmic domain of VAMP2. Munc18a was incubated with t-SNARE-T14E for 1 h on ice, and next, v-SNARE was added. The addition of CDV was first, and then Munc18a was added for a negative control. (**B**,**C**) Kinetic fusion graph of unlabeled acceptor t-SNARE and mutant t-SNARE-T14E proteoliposome (1.5 µM) mixed with fluorescently labeled donor v-SNARE proteoliposome (1.5 µM) in the presence of 10 µM Munc18a or an equal volume of buffer (A100). Background fusion was measured by the addition of the cytoplasmic domain of VAMP2 (CDV) to a final concentration of 20 µM (negative control). The fusion assay was performed for t+v-SNAREs, t+v-SNAREs+Munc18a, t+v-SNAREs+CDV, and t+v-SNAREs+CDV+Munc18a at 37 °C for 2 h after incubating overnight at 4 °C. Data are represented as percent (%) of maximum fluorescence versus time (minute). The total volume of all the fusion setups was brought up to a final volume of 60 µL. (**D**) Fusion reaction rates for t+v-SNAREs and t-v-SNAREs+Munc18a were presented as a bar graph of the reconstituted fusion assay shown in (**B**,**C**). Data presented as the percentage of fluorescence change per 120 min. Error bars indicate standard error mean. Data presented as mean ± SEM (*n* = 4 independent replicates). *p* values were calculated using a Student *t*-test. *** *p* < 0.002.

Figure 3 shows the effect of including a ten-fold molar excess (15 µM) of recombinant Munc18a. While Munc18a substantially stimulated membrane fusion with syntaxin1A [21,24], Munc18a produced a small, barely significant improvement in fusion mediated by SNARE complexes containing wild-type syntaxin3B when compared to wild-type syntaxin3B-containing SNARE complexes alone (compare Figure 1D and Figure 3D, blue bars; without Munc18: 4.92 ± 0.15; with Munc18: 5.76 ± 0.18; *n* = 4; *p* = 0.0471; one-way ANOVA followed by Tukey’s multiple comparisons). By contrast, fusion driven by the syntaxin3B(T14E) in the presence of Munc18a was significantly enhanced relative to wild-type syntaxin3B with Munc18a (Figure 3C,D). This result is likely the mixture of the subtle improvement afforded by syntaxin3B(T14E) alone and the improvement contributed by the presence of Munc18a (compare also Figure 1D and Figure 3D, red bars; STX3B-T14E without Munc18: 6.04 ± 0.20; STX3B-T14E with Munc18: 7.73 ± 0.25; *n* = 4; *p* = 0.0003; one-way ANOVA followed by Tukey’s multiple comparisons).

### 3.4. Complexin III and Complexin IV Regulate Membrane Fusion Catalyzed by Syntaxin3B-Containing SNARE Complexes

**Figure 4:**
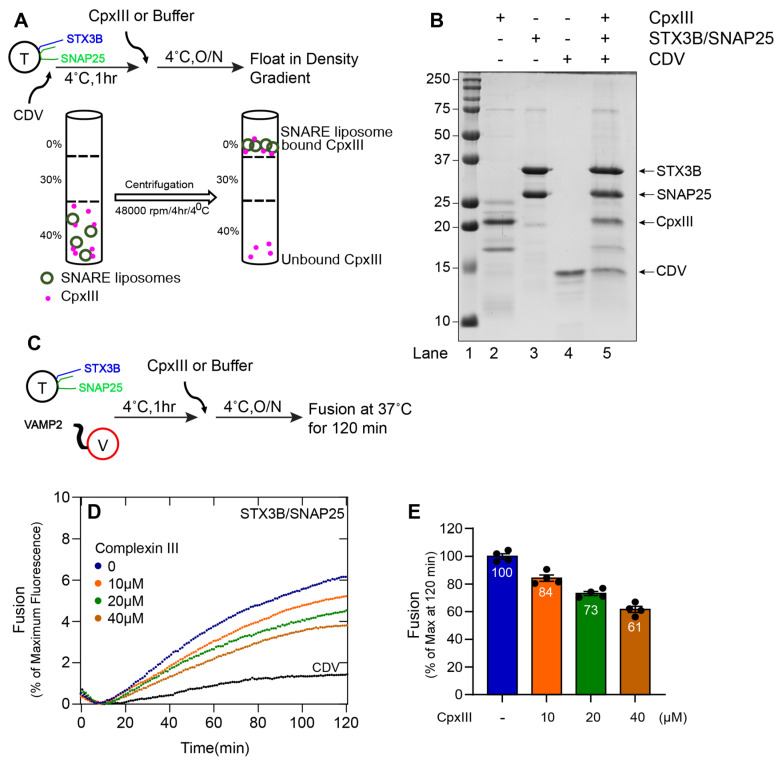
Complexin III functionally interacts with the assembled (Syntaxin3B/SNAP25b) t-SNARE complex and inhibits membrane fusion. (**A**) A schematic representation of Complexin III (CpxIII) binding to liposome containing tertiary retinal/exocytic SNARE complexes was prepared by incubating the wild-type t-SNARE complex (Syntaxin3B/SNAP25, here indicated as T) (5 µM) with soluble VAMP2 (CDV) (20 µM) overnight at 4 °C. Accudenz gradient preparation (40%, 30%, and 0%) and centrifugation were performed to obtain CpxIII-bound tertiary retinal/exocytic SNARE complexes. (**B**) Coomassie blue-stained SDS-PAGE gel showing the binding of CpxIII to tertiary retinal/exocytic SNARE liposome. Lane 1, Mr = Marker. Lanes 2–4, input materials. Lane 5, the float-up result showing the binding of CpxIII to indicated tertiary retinal/exocytic SNARE liposome. Ten microliters of sample were loaded per lane on 15% SDS-PAGE. The arrow indicates the respective protein band with its molecular weight. (**C**) A schematic representation of the incubation method for t-SNARE, v-SNARE (here indicated as V), and CpxIII. Where t-SNARE and v-SNARE were mixed with each other and incubated for 1 h, and CpxIII was added and incubated at 4 °C overnight. Background fusion was measured by the addition of the cytoplasmic domain of VAMP2 (CDV) to a final concentration of 20 µM (negative control). (**D**) The fusion assay was performed for T+V, T+V+CpxIII (10 µM), T+V+CpxIII (20 µM), T+V+CpxIII (40 µM) and T+V+CDV. Proteoliposomes T (1.5 µM) and V (1.5 µM) concentration and CDV at 20 µM. The fusion assay was performed at 37 °C for 2 h. Data are represented as percent (%) of maximum fluorescence versus time (minute). The total volume of all the fusion setups was brought up to a final volume of 60 µL. (**E**) The extent of fusion at 120 min was compared to results with buffer control and presented as a percent of the control measurement. Each dot represents the independent fusion experiments (*n* = 4). Data presented as mean ± SEM.

**Figure 5:**
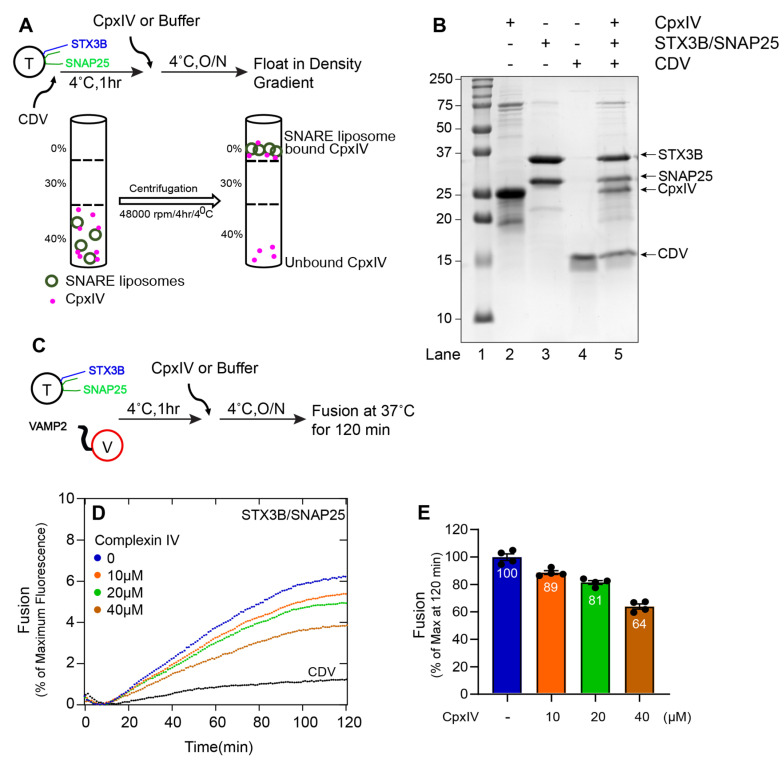
Complexin IV functionally interacts with the assembled (Syntaxin3B/SNAP25B) SNARE complex and inhibits membrane fusion. (**A**) A schematic representation of Complexin IV (CpxIV) binding to liposome containing tertiary retinal/exocytic SNARE complexes was prepared by incubating the wild-type t-SNARE complex (Syntaxin3B/SNAP25, here indicated as T) (5 µM) with soluble VAMP2 (CDV) (20 µM) overnight at 4 °C. Accudenz gradient preparation (40%, 30%, and 0%) and centrifugation were performed to obtain CpxIV-bound tertiary retinal/exocytic SNARE complexes. (**B**) Coomassie blue-stained SDS-PAGE gel showing the binding of CpxIV to tertiary retinal/exocytic SNARE liposome. Lane 1, Mr = Marker. Lanes 2–4, input materials. Lane 5, float-up result showing the binding of CpxIV to indicated tertiary retinal/exocytic SNARE liposome. Ten microliters of sample were loaded per lane on 15% SDS-PAGE. The arrow indicates the respective protein band with its molecular weight. (**C**) A schematic representation of the fusion assay incubation method for t-SNARE, v-SNARE (here indicated as V), and CpxIV. Where t-SNARE and v-SNARE were mixed with each other and incubated for 1 h. Next, CpxIV was added and incubated at 4 °C overnight. Background fusion was measured by the addition of the cytoplasmic domain of VAMP2 (CDV) to a final concentration of 20 µM (negative control). (**D**) The fusion assay was performed for T+V, T+V+CpxIV (10 µM), T+V+CpxIV (20 µM), T+V+CpxIV (40 µM) and T+V+CDV. Proteoliposomes T (1.5 µM) and V (1.5 µM) concentration and CDV at 20 µM. The fusion assay was performed at 37 °C for 2 h. Data are represented as percent (%) of maximum fluorescence versus time (minute). The total volume of all the fusion setups was brought up to a final volume of 60 µL. (**E**) The extent of fusion at 120 min was compared to results with buffer control and presented as a percent of the control measurement. Each dot represents the independent fusion experiments (*n* = 4). Data presented as mean ± SEM.

We next sought to clarify the role of Cpx III and Cpx IV on membrane fusion catalyzed by syntaxin3B-containing SNARE complexes. We expressed and purified both recombinant mouse Cpx III and Cpx IV in *E. Coli*. Using a liposome co-flotation assay, we found that both Cpx III (Figure 4A,B) and Cpx IV (Figure 5A,B) bound to the fully assembled cis-SNARE complexes formed between syntaxin3B, SNAP25, and soluble VAMP2 (CDV).

We then asked whether Cpx III and Cpx IV regulate membrane fusion. Previously, we have shown that Drosophila complexin inhibits in vitro membrane fusion with canonical neuronal SNAREs at hemifusion [36]. When either Cpx III (Figure 4D,E) or Cpx IV (Figure 5D,E) was incubated with syntaxin3B-containing t-SNARE proteoliposomes and v-SNARE proteoliposomes, lipid mixing was reduced in a concentration-dependent manner. These results confirm that retinal ribbon synapse SNARE proteins are susceptible to regulation by the complexin proteins expressed by retinal ribbon synapses.

## 4. Discussion

In this study, we examined the regulation of membrane fusion catalyzed by syntaxin3B-containing SNARE complexes under conditions that allowed for the precise control of protein and lipid composition. The mechanisms described here are not only applicable to syntaxin3B-expressing synapses in the retina but also to other cells that may utilize syntaxin3B. In addition, the N-terminal domain through the first half of the SNARE binding domain of syntaxin3B is identical to that of syntaxin3A, the ubiquitous syntaxin 3 splice form [3]. Therefore, the regulatory mechanisms involving the N-terminal domain described here may be conserved between these two syntaxin 3 splice variants and impact syntaxin3-mediated membrane fusion events elsewhere in the body, including in the immune, renal, endocrine, and digestive systems [6,53,54,55,56,57,58,59].

In this study, we demonstrate for the first time that a phosphomimetic mutation of syntaxin3B at T14 promotes SNARE-mediated membrane fusion. While the effect was quite modest, it is important to note that in our experiments, syntaxin3B and SNAP25 were co-expressed and incorporated together into the acceptor liposome. These two proteins had most likely preassembled into a t-SNARE complex prior to the addition of the VAMP2-containing liposome and the start of our measurements, and therefore, a faciliatory effect of the T14 phosphomimetic mutant on the conversion of closed syntaxin3B to open syntaxin3B [9,13] would not be captured by our assay. For this reason, the present results should be taken as a lower limit of the impact of T14s phosphorylation status on membrane fusion mediated by syntaxin3B-containing SNARE complexes.

Munc18 has two main roles in SNARE-mediated membrane fusion: stabilizing the syntaxin closed conformation and nucleating SNARE complex formation. Here, we examined the role of Munc18 downstream of t-SNARE complex formation. As anticipated, Munc18a facilitated membrane fusion mediated by syntaxin3B-containing SNARE complexes, although to a far lesser extent than has been reported for syntaxin1A [21,24]. This could be due to a differential effect of Munc18a on syntaxin3B-mediated fusion versus syntaxin1A-mediated fusion. However, there are also significant methodological differences between the two studies that could also contribute to this difference. Munc18a bound equally well to SNARE complexes containing wild-type syntaxin3B or syntaxin3B with the T14E mutation, consistent with an earlier report from our group [9]. However, there was a differential effect on membrane fusion. Munc18a had a greater stimulatory effect on fusion mediated by syntaxin3B with the T14E mutation than on wild-type. Furthermore, the combination of T14E syntaxin 3 mutant and Munc18a together produced the most membrane fusion. These results suggest an additional modulatory effect of T14 that is downstream of its ability to facilitate the transition of syntaxin3B from a closed to an open conformation [9,13]. While we focused on Munc18a and syntaxin3B in this study, we note that outside of the retina, syntaxin3A may work with Munc18b to regulate membrane fusion [30,53,60].

Using our carefully-controlled in vitro fusion assay, we established that Cpxs III and IV inhibit membrane fusion mediated by syntaxin3B-containing SNARE complexes and that they do so in a dose dependent manner. This inhibition may occur by regulating hemifusion arrest, as has been shown previously for syntaxin1A [36], although other actions, such as preventing full SNARE complex assembly, might also contribute [61,62,63]. In general, CPXs III and IV have been reported to suppress calcium-independent release and facilitate evoked release [40,41,42], similar to the actions of complexins I and II at conventional synapses [51,61,64,65,66,67,68]. Our results provide a mechanistic explanation that can account for these actions in the retina. By clamping the syntaxin3B-containing SNARE complexes until the arrival of an appropriate calcium stimulus, Cpxs III and IV will suppress release, thereby reducing synaptic noise, and enhance the gain of retinal ribbon-style synapses.

## Data Availability

All of the data is contained within the figures. The primary, unprocessed data is available from the authors upon request.
